# An Evaluation of VYC‐17.5L for the Treatment of Marionette Lines: A Prospective, Open‐Label, Postmarketing Study

**DOI:** 10.1111/jocd.16694

**Published:** 2024-11-27

**Authors:** Sofia Ruiz Del Cueto, Fernando Urdiales Galvez, Alessandro Gritti, Nicola Kefalas, Carola de la Guardia, Graeme Kerson

**Affiliations:** ^1^ Clínica Mira and Cueto Madrid Spain; ^2^ Instituto Médico Miramar Málaga Spain; ^3^ GRITTI Chirurgia Maxillo Facciale Milan Italy; ^4^ Private Practice Torino Italy; ^5^ Allergan Aesthetics, an AbbVie Company Madrid Spain; ^6^ Allergan Aesthetics, an AbbVie Company Bucks UK

**Keywords:** facial wrinkles, hyaluronic acid, marionette lines, oral commissures, VYC‐17.5 L

## Abstract

**Background:**

Marionette lines are a feature of the aging face, descending from the oral commissures towards the jaw. VYC‐17.5 L is a dermal filler that contains 17.5 mg/mL of hyaluronic acid (HA) and lidocaine (3 mg/mL); it is intended for the treatment of skin depressions. VYC‐17.5 L has been shown to be safe and effective in different conditions, but there is a lack of published literature on its effectiveness in marionette lines.

**Aims:**

This 12‐month prospective, open‐label, post‐marketing study evaluated the effectiveness and safety of the injectable HA filler VYC‐17.5 L for the improvement of marionette lines.

**Methods:**

Adults (≥ 18 years) with mild‐to‐severe marionette lines on the validated Allergan Marionette Line Scale (AMLS) received VYC‐17.5 L on Day 1 with optional touch‐up on Day 14. The primary endpoint was proportion of participants with ≥ 1‐point change in AMLS from baseline at Month 1 (M1). Secondary endpoints were investigator‐ and participant‐rated Global Aesthetic Improvement Scale (GAIS), FACE‐Q satisfaction with facial appearance, and appraisal of lines: marionette. Safety was assessed throughout.

**Results:**

A total of 83 participants completed the study; 69.9% of participants had ≥ 1‐point change in AMLS at M1. Investigator and participant GAIS showed improvement. Both FACE‐Q scores significantly improved from baseline (*p* < 0.0001). A significant volume improvement was seen and maximized at M1. Most participant‐reported injection site reactions were mild or moderate and resolved within 8 days; 14 subjects reported adverse device effects, with the most common being pain, which resolved within 8 days.

**Conclusion:**

This prospective, open‐label study showed that VYC‐17.5 L effectively improved marionette lines and was well tolerated.

## 1 Introduction

The lower third of the face is one of the most prominent areas to show signs of aging. Aging of the perioral region because of bone resorption, gravity, contraction of the oral musculature, and loss of collagen and elastin in the skin leads to the appearance of melomental folds or marionette lines, a hallmark of facial aging [1, 2]. The marionette lines descend from the oral commissures towards the jaw; the superior portion of marionette lines is formed by the cutaneous insertion of the depressor anguli oris muscle, while the inferior portion comprises the mandibular ligament [1, 2].

The injection of hyaluronic acid (HA) dermal fillers is a common and minimally invasive approach for the treatment of several anatomical areas of the face including nasolabial folds and radial cheek lines [3, 4]. Treatment of the marionette lines via dermal fillers is the most common approach despite the lack of published clinical data specific to this indication. Carruthers and colleagues conducted a 24‐week assessment of HA filler in combination with onabotulinum toxin A in the lips and marionette lines and found that treatment was effective, but no details specific to the marionette lines were given [5]. Previous studies have investigated the effectiveness of HA fillers when used across several regions of the face at once. In a 6‐month study investigating a range of HA fillers in various regions of the face, Rzany and colleagues reported that the marionette lines were the second most‐treated area and patients were highly satisfied with the aesthetic improvement, but the volume of filler used was quite large [6]. Thus, clinical studies focused on the treatment of marionette lines at smaller volumes are needed.

Juvederm VOLIFT with lidocaine (VYC‐17.5 L; Allergan Aesthetics, an AbbVie Company) is a soft tissue filler containing 17.5 mL of HA and 3 mg/mL of lidocaine that is intended for the treatment of skin depressions due to premature aging. VYC‐17.5 L is manufactured with Vycross technology using a patented mix of long (> 500 kDa) and longer (> 1 MDa) HA chains and was designed with tailored rheologic and physicochemical characteristics (such as elasticity, cohesivity, and water uptake potential) for the intended indication. VYC‐17.5 L is a smooth, moldable homogeneous gel with moderate lift capacity and good tissue integration [3]. The safety and efficacy of VYC‐17.5 L has been demonstrated in several anatomical locations, but little has been published on its effectiveness for treating marionette lines [3, 4]. Thus, this study aimed to evaluate the safety and efficacy of VYC‐17.5 L for the treatment of marionette lines.

## 2 Materials and Methods

### 2.1 Study Design

**FIGURE 1:**
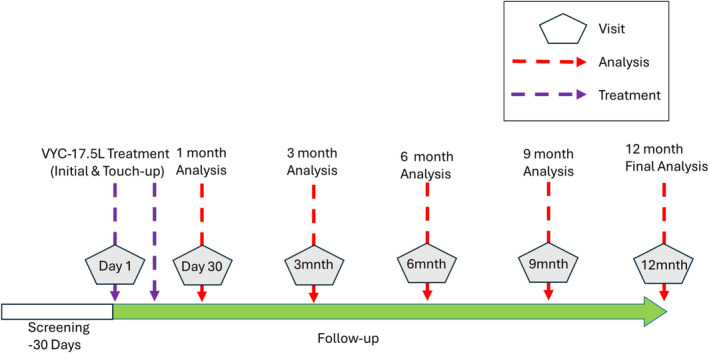
Study design schema.

This was a 12‐month, prospective, open‐label, single‐group, postmarketing study conducted at one site in France from September 2021 through November 2022. This study conformed to the ethical guidelines of the 1975 Declaration of Helsinki. Independent ethics committee (IEC) approval was obtained from the Comité de Protection des Personnes (Lyon, France). All participants provided informed consent prior to any study‐related procedures being performed. This study comprised up to seven visits: screening (Day −30 to Day 0), baseline visit (Day 1), an optional touch‐up visit (Day 14), and follow‐up thereafter for 12 months (at Months 1, 3, 6, 9, and 12; Figure 1).

### 2.2 Participants

Eligible participants were adults (≥ 18 years of age) who had mild‐to‐very severe (Grades 1–4) marionette lines on both sides based on the validated Allergan Marionette Line Scale (AMLS; scale of 0–4; 0 = none, 1 = minimal, 2 = moderate, 3 = severe, and 4 = very severe). Additional inclusion criteria included: no other procedures or treatments anywhere in the face not related to the study, negative pregnancy tests before each treatment for women of childbearing potential, and affiliation with a health social system.

Participants were not eligible if they were pregnant or nursing female participants or female participants who plan to get pregnant during the duration of the study; had undergone facial plastic surgery or received permanent facial implants anywhere in the face; had undergone cosmetic resurfacing (i.e., laser, photomodulation, intense pulsed light radiofrequency, dermabrasion, and chemical peel) anywhere on the face or neck in the last 6 months; used any topical, oral, over‐the‐counter or prescription antiwrinkle products within 90 days of enrollment; any ongoing regimen of anticoagulation therapy (e.g., warfarin) or nonsteroidal anti‐inflammatory drugs within 10 days of study treatment; or had an active infection, inflammation (acne, herpes, and cancerous or precancerous lesion), or unhealed wound.

### 2.3 Treatment

Treatment was administered following completion of screening, enrollment, and obtaining informed consent from each participant. On Day 1, participants were injected in the marionette lines at a dosage and injection pattern determined by the specialist injector (SI) based on their clinical experience. The majority of investigators used a combination of fanning and linear threading techniques. The maximum volume was 2 mL for each marionette line for both the initial and touch‐up visit combined, with a 1 mL allowable volume per marionette line per treatment session. On Day 14, an optional touch‐up treatment was provided if the investigator and participant determined it was necessary based on the aesthetic results of the first injection. The primary evaluation of effectiveness occurred in Month 1 (M1).

### 2.4 Efficacy Endpoints

The primary efficacy endpoint was achievement of ≥ 1‐point improvement on the validated AMLS from baseline at M1 for both the right and left marionette lines. The investigator assessed the right and left marionette lines using the validated AMLS. The AMLS is a validated 5‐point photonumeric scale for the investigator to assess a participant's marionette line severity [7]. The AMLS includes five grades (0 = none, 1 = minimal, 2 = moderate, 3 = severe, and 4 = very severe) and was used to evaluate both sides of the lower face at each assessment timepoint. Secondary efficacy endpoints included the following: proportion of participants who achieve ≥ 1‐point improvement on the AMLS from baseline at Months 3, 6, 9, and 12; change in Rasch transformed overall FACE‐Q Satisfaction with facial appearance/appraisal of lines scores from baseline to Months 1, 3, 6, 9, and 12; proportion of investigator and participant ratings of “improved” or “much improved” on the Global Aesthetic Improvement Scale (GAIS) using baseline two‐dimensional (2D) photographs at months 1, 3, 6, 9, and 12; and volume change in marionette lines from baseline to all timepoints via three‐dimensional (3D) imaging using the Vectra M3 Lift 3D System (Canfield Scientific; Parsippany, New Jersey). The Vectra M3 is a photography system that uses high resolution to capture a detailed 3D image of the participant's face. The GAIS utilizes a 5‐point Likert scale (scale of 2 to −2: 2 = much improved, 1 = improved, 0 = no change, −1 = worse, and −2 = much worse; Table 1) to assess the improvement in marionette line appearance. Scores from each FACE‐Q questionnaire were summed and converted, known as Rasch transformation, into a score from 1 to 100 to permit interpretation.

### 2.5 Safety Endpoints

Safety, including the incidence and severity of adverse events (AEs) were monitored throughout the study. Patient diaries were used to capture injection site reactions (ISRs) for 30 days and to assess the tolerance of VYC‐17.5 L on a 4‐point photonumeric scale (scale of 0–3; 0 = none, 1 = mild, 2 = moderate, and 3 = severe). ISRs were not reported as AEs unless they were still present 30 days after injection, required medical intervention, or were considered by the investigator to be abnormal.

### 2.6 Statistical Analyses

Based on a previous study with a participant response rate of 89% and assuming a 10% attrition rate, approximately 80 participants would produce a two‐sided 95% confidence interval (CI) with a width of 0.15 [3]. The modified intention‐to‐treat (mITT) population consisted of all participants with at least one baseline and postbaseline marionette lines assessment. Efficacy analyses were performed on the mITT population. The change in volume from baseline was analyzed using paired *t*‐tests. All safety analyses were performed on the safety population, which consisted of all treated participants. All statistical tests were two‐sided hypothesis tests performed at the 5% significance level using SAS software version 9.4.7.

**TABLE 1:** Investigator and participant global aesthetic improvement scale.

Participant description	Score	Grade	Investigator description
No description provided; only comparison to baseline photo	2	Much improved	Marked improvement in appearance
	1	Improved	Improvement in appearance, but a touch‐up or retreatment is indicated
	0	No Change	The appearance is essentially the same as the original condition
	–1	Worse	The appearance is worse than the original condition
	−2	Much Worse	The appearance is much worse than the original condition

## 3 Results

### 3.1 Participants

Of 130 screened participants, 83 were enrolled, and 78 (94%) completed the 12‐month study. Reasons for discontinuing included withdrawal by the participant (*n* = 3), lost to follow‐up (*n* = 1), and an AE (*n* = 1). The mean age of participants was 59.2 years. Most participants were female (95.2%), with Fitzpatrick skin phototypes I–IV represented (Table 2). The average injection volume was 0.73 mL for the initial treatment and 0.35 mL for those participants (*n* = 54; 65.1%) who received a touch‐up injection.

**TABLE 2:** Participant demographics (safety population).

Characteristic	Marionette lines (*n* = 83)
Age, years	
Mean (SD)	59.2 (8.2)
Median	60.0
Range	34–70
Sex, *n* (%)	
Female	79 (95.2)
Male	4 (4.8)
Fitzpatrick skin phototype, *n* (%)	
I	1 (1.2)
II	33 (39.8)
III	33 (39.8)
IV	16 (19.3)

*Note:* Participants were treated at a single site in France.

Abbreviation: SD, standard deviation.

### 3.2 Efficacy: Investigator AMLS, and GAIS and FACE‐Q Scores

**FIGURE 2:**
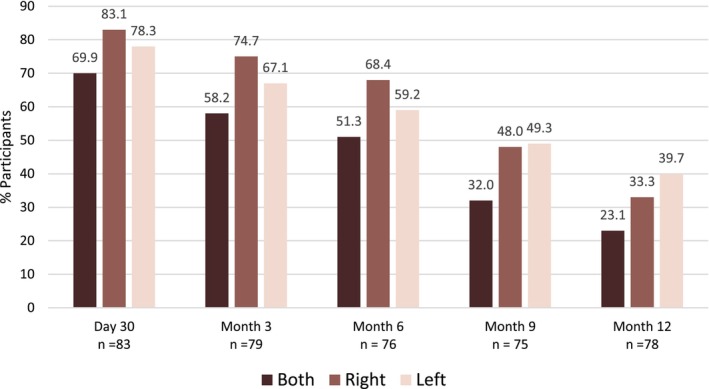
Proportion of responders achieving investigator‐assessed AMLS ≥ 1‐Point improvement from baseline over 12 months. Both the right and left marionette lines were assessed; primary endpoint required a change on the right and left marionette lines on the AMLS. AMLS, Allergan Marionette Line Scale.

The distribution of baseline AMLS among participants was as follows: 1% (Grade 0), 19% (Grade 1), 36% (Grade 2), 35% (Grade 3), and 10% (Grade 4). The proportion of participants within each AMLS severity category throughout the study is noted in Table 3. The primary endpoint showed 69.9% of participants demonstrating a ≥ 1‐point improvement from baseline at M1 on the investigator‐assessed AMLS for the right and left marionette lines (Figure 2). A measured decline in response was observed at Months 3 and 6 (58.2% and 51.3%, respectively) on the AMLS and continued through Month 12 following treatment with VYC‐17.5 L (Figure 2).

**TABLE 3:** Proportion of participants within each AMLS category over the course of the study.

	AMLS score	
	Right ML	Left ML
Baseline, *N* (%)		
*N* (MV)	83 (0)	83 (0)
None	0 (0.0)	1 (1.2)
Minimal	18 (21.7)	14 (16.9)
Moderate	30 (36.1)	29 (34.9)
Severe	29 (34.9)	29 (34.9)
Very Severe	6 (7.2)	10 (12.0)
Month 1, *N* (%)		
*N* (MV)	83 (0)	83 (0)
None	10 (12.0)	8 (9.6)
Minimal	53 (63.9)	44 (53.0)
Moderate	18 (21.7)	27 (32.5)
Severe	2 (2.4)	4 (4.8)

Very Severe	0 (0.0)	0 (0.0)
Month 3, *N* (%)		
*N* (MV)	79 (4)	79 (4)
None	10 (12.7)	7 (8.9)
Minimal	41 (51.9)	35 (44.3)
Moderate	26 (32.9)	34 (43.0)
Severe	2 (2.5)	3 (3.8)
Very Severe	0 (0.0)	0 (0.0)
Month 6, *N* (%)		
*N* (MV)	76 (7)	76 (7)
None	4 (5.3)	2 (2.6)
Minimal	37 (48.7)	28 (36.8)
Moderate	30 (39.5)	39 (51.3)
Severe	5 (6.6)	6 (7.9)
Very Severe	0 (0.0)	1 (1.3)
Month 9, *N* (%)		
*N* (MV)	75 (3)	75 (3)
None	3 (4.0)	3 (4.0)
Minimal	26 (34.7)	21 (28.0)
Moderate	33 (44.0)	35 (46.7)
Severe	11 (14.7)	15 (20.0)
Very Severe	2 (2.7)	1 (1.3)
Month 12, *N* (%)		
*N* (MV)	78 (5)	78 (5)
None	2 (2.6)	2 (2.6)
Minimal	23 (29.5)	19 (24.4)
Moderate	35 (44.9)	35 (44.9)
Severe	15 (19.2)	20 (25.6)
Very Severe	3 (3.8)	2 (2.6)

Abbreviation: MV, missing value.

**FIGURE 3:**
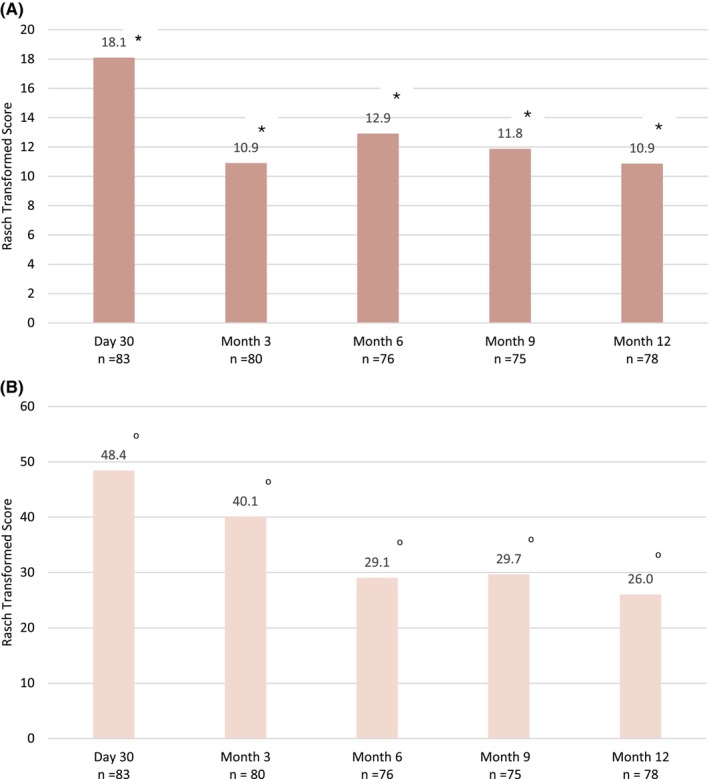
FACE‐Q appraisal of lines (A) and satisfaction with facial appearance (B) change from baseline scores over 12 months following treatment with VYC‐17.5 L. ^o^ *p* < 0.0001 for the paired Student's *t*‐test (FACE‐Q appraisal of lines); *p < 0.0001 for the Wilcoxon test (satisfaction with facial appearance).

The mean change in the Rasch transformed FACE‐Q satisfaction with facial appearance total score from baseline was 18.1 at M1 (*p* < 0.0001; Figure 3A). The mean change in total score decreased over time from 18.1 to 10.9 at M12. Most participants were somewhat or very satisfied with the proportionality, balance, and freshness of their face 1 month after treatment with VYC‐17.5 L, with a downward trend emerging from M3 to M12. In a similar fashion, the Rasch transformed FACE‐Q appraisal of lines mean change from baseline score ranged from 48.4 at M1 to 26.0 at M12 (*p* < 0.0001 for all timepoints; Figure 3B).

**FIGURE 4:**
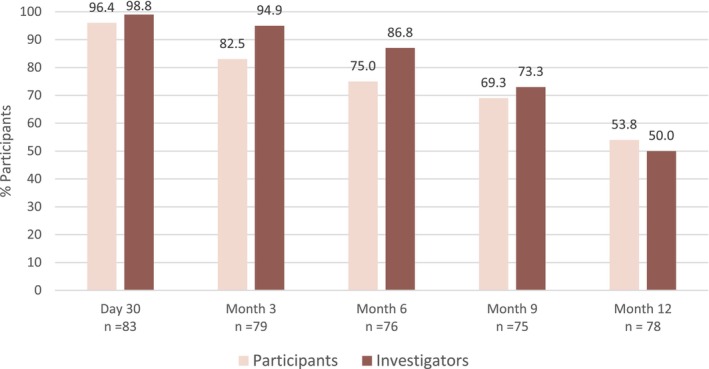
Proportion of responders achieving investigator‐ and participant‐assessed GAIS rating of improved or much improved over the course of the study. GAIS, Global Aesthetic Improvement Scale.

**FIGURE 5:**
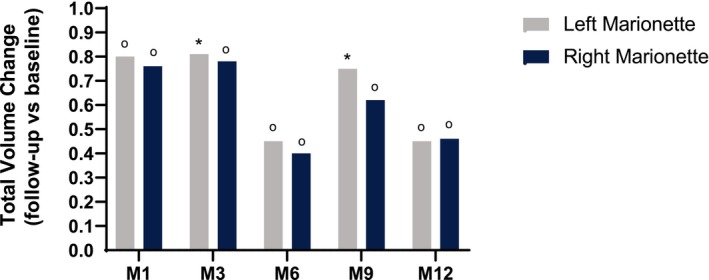
Change in total volume from baseline in participants throughout the course of the study after treatment with VYC‐17.5 L. M1, 3, 6, 9, and 12 = Months 1, 3, 6, 9, and 12; *p < 0.05 for the Wilcoxon test; ^o^ *p* < 0.05 for the paired Student's *t*‐test.

**FIGURE 6:**
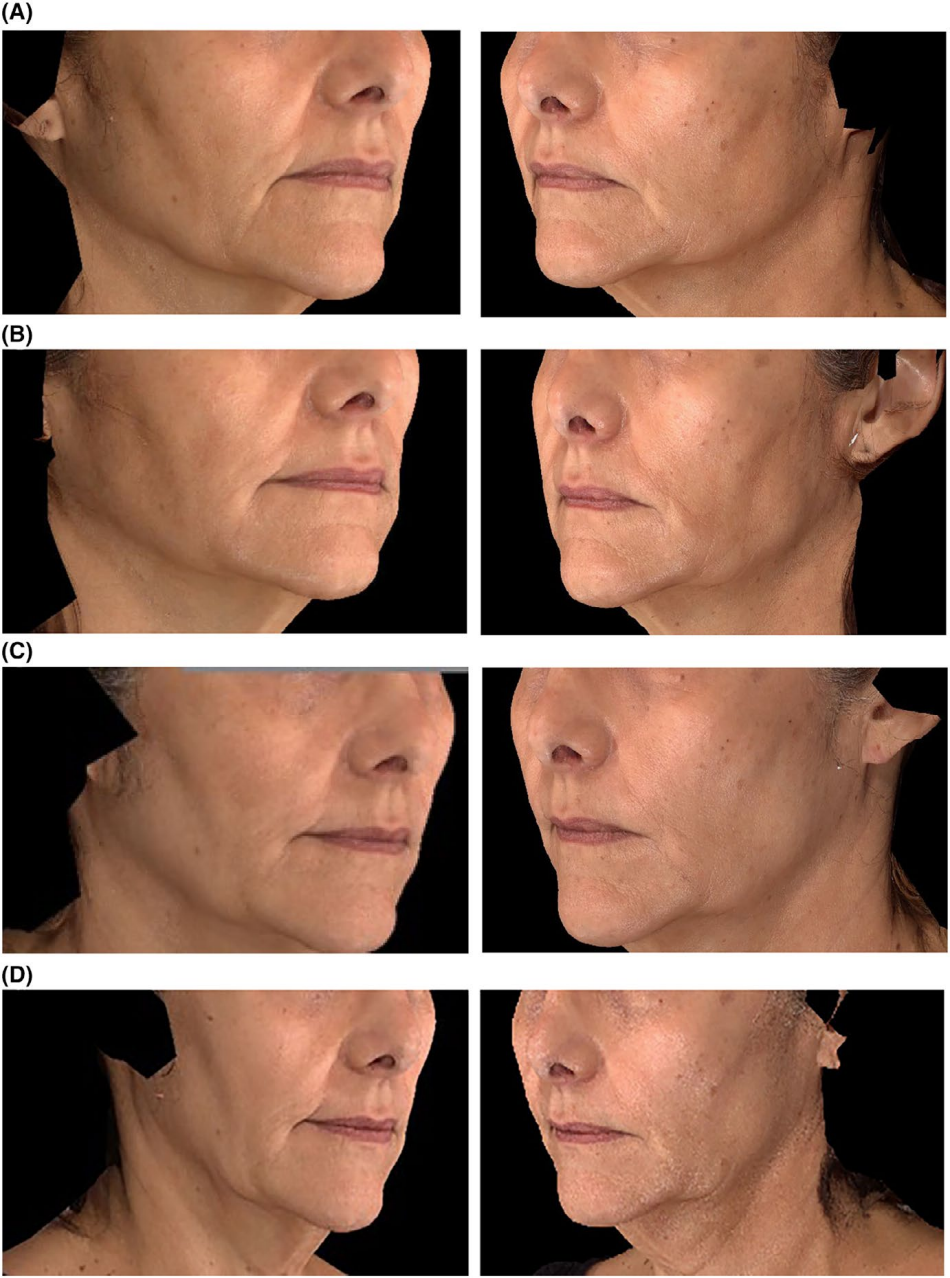
Representative photos of ≥ 1‐point improvement on the AMLS on Day 1 and Month 1 after treatment with VYC‐17.5 L. Baseline (grade: severe) and Month 1 (grade: minimal) photos of a 57‐year‐old participant. Approximately 1 mL of VYC‐17.5 L was injected into the right ML and 0.8 mL was injected into the left ML. AMLS, Allergan Marionette Line Scale; ML, Marionette line.

The proportion of participants achieving a rating of “improved” or “much improved” on the investigator‐assessed GAIS was 98.8% at M1 and subsequently declined to 50% over the following 11 months (Figure 4). The participant‐assessed GAIS mirrored that of the investigator GAIS with 96.4% of participants rating the appearance of their marionette lines as “improved” or “much improved” at M1. The rating slowly declined at subsequent follow‐up visits from 82.5% at M3 to 53.8% at M12 (Figure 4). Lastly, a significant change in total volume in the right and left marionette lines was seen at M1 compared to baseline (0.80 and 0.76, respectively; *p* < 0.0001; Figure 5) before trending downward from M3 through M12. Representative photos of improvement in ML over the course of the study are shown in Figure 6.

### 3.3 Safety

The most common ISRs were pain/tenderness, swelling/edema, lumps/bumps, induration, redness/erythema, bruising/hematoma, itching, and discoloration/hyperpigmentation. The most frequently reported ISRs were pain/tenderness with 80% of participants experiencing this response. The majority of ISRs were mild in severity, lasted 1–3 days, and resolved spontaneously. Fourteen adverse device effects (ADEs) were reported in 13 participants. The most common ADE was pain (50%) followed by mass and nodules (14%, respectively; Table 4). Most ADEs lasted less than 8 days and resolved spontaneously. In addition, no serious ADEs were reported.

**TABLE 4:** Summary of ADEs occurring among participants treated with VYC‐17.5 L.

ADE Category	Marionette lines (*n* = 14)
Participants with at least 1 ADE, *n* (%)	14 (100.0)
General disorders and administration site conditions, *n* (%)	
Injection site hematoma	1 (7.1)
Injection site irritation	1 (7.1)
Injection site mass	2 (14.3%)
Injection site nodule	2 (14.3%)
Injection site pain	7 (50.0)
Injection site urticaria	1 (7.1%)

Abbreviation: ADEs, adverse device effects.

## 4 Discussion

This single‐center, 12‐month postmarketing study showed significant improvement in marionette lines from baseline after treatment with up to 2 mL of VYC‐17.5 L. Improvements were observed in investigator and participant assessments and via photography‐based measurement. High participant ratings of satisfaction with VYC17.5 L treatment were reported throughout the study. ISR and ADEs related to treatment with VYC‐17.5 L were expected, primarily local and resolved on their own.

Previous studies have evaluated the impact of HA fillers. The US HARMONY study evaluated several HA fillers across several areas of the face and reported high patient satisfaction on the FACE‐Q satisfaction with facial appearance scale and a good safety profile [8]. Solish and colleagues evaluated the naturalness of a variety of HA dermal fillers in different anatomical areas of the face, including the marionette lines, and found that the severity of the marionette lines was significantly improved, although the cohort being assessed was small and homogenous [9].

It is worth noting that based on the validated AMLS, not all patients may need a 1‐point improvement of their marionette lines. Some participants in the study did not achieve the primary endpoint (i.e., a 1‐point improvement on the AMLS) but were still satisfied with the treatment based on the patient‐reported GAIS and FACE‐Q satisfaction ratings. Thus, even small improvements in the appearance of marionette lines may result in high patient satisfaction.

One potential limitation of this study is the lack of diversity among participants; most participants had a Fitzpatrick skin phototype of II or III. In addition, the majority of participants (92.8%) in the study identified as female. Marionette lines also occur in men and the number of male participants in the study was low, limiting the generalizability of the results.

## 5 Conclusion

Treatment of marionette lines with VYC‐17.5 L is effective and well tolerated for up to 12 months.

## Author Contributions

All authors contributed to the conception and design of the paper and the interpretations provided; were involved in drafting the manuscript and revising it critically for important intellectual content; gave final approval of the version to be published; and agreed to be accountable for all aspects of the work.

## Ethics Statement

The authors confirm that independent ethics committee approval was obtained from Comite de Protection des Personnes (Lyon, France). Participants provided informed consent prior to the initiation of study procedures during the screening visit.

## Conflicts of Interest

Sofia Ruiz Del Cueto: The author declares no conflicts of interest. Fernando Urdiales Galvez: Investigator for Allergan Aesthetics, an AbbVie company. Alessandro Gritti: Advisory board member, speaker, and trainer for Allergan Aesthetics, an AbbVie company. Nicola Kefalas: Advisory board member, speaker, and trainer for Allergan Aesthetics, an AbbVie company. Carola de la Guardia: Employee of AbbVie and may own AbbVie stock/options. Graeme Kerson: Employee of AbbVie and may own AbbVie stock/options.

## Data Availability

AbbVie is committed to responsible data sharing regarding the clinical trials we sponsor. This includes access to anonymized, individual, and trial‐level data (analysis data sets), as well as other information (eg, protocols, clinical study reports, or analysis plans), as long as the trials are not part of an ongoing or planned regulatory submission. This includes requests for clinical trial data for unlicensed products and indications. These clinical trial data can be requested by any qualified researchers who engage in rigorous, independent, scientific research, and will be provided following review and approval of a research proposal, Statistical Analysis Plan (SAP), and execution of a Data Sharing Agreement (DSA). Data requests can be submitted at any time after approval in the US and Europe and after acceptance of this manuscript for publication. The data will be accessible for 12 months, with possible extensions considered. For more information on the process or to submit a request, visit the following link, then selct "Home": https://vivli.org/ourmember/abbvie/.
